# A case report of insulin autoimmune syndrome induced by drugs for colorectal cancer

**DOI:** 10.1097/MD.0000000000045456

**Published:** 2025-11-14

**Authors:** Xianglei Wu, Leilei Bao, Guorong Fan, Shuowen Wang

**Affiliations:** a Department of Clinical Pharmacy, Shanghai General Hospital, Shanghai Jiao Tong University School of Medicine, Shanghai, China; b Department of Pharmacy, Third Affiliated Hospital of Naval Medical University, Shanghai, China.

**Keywords:** adverse drug reaction, case report, IAS, omeprazole, oxaliplatin

## Abstract

**Rationale:**

Insulin autoimmune syndrome (IAS) is a rare autoimmune endocrine disease. At present, there are no recognized guidelines. We present a case study of a colorectal cancer patient who was diagnosed with IAS following standard postoperative adjuvant chemotherapy. After comprehensive intervention with glucose supplementation and glucocorticoid therapy successfully normalized the patient’s blood glucose levels. This case highlights a potential association between widely prescribed proton pump inhibitors and oxaliplatin chemotherapy with IAS development. These findings emphasize the importance of clinical vigilance for IAS in patients presenting with hypoglycemia.

**Patient concerns:**

A 70-year-old Chinese male, after colon cancer surgery, has been regularly receiving antitumor treatment with bevacizumab combined with the FOLFOX regimen. After the 8th cycle of this regimen, he developed symptoms of palpitations and cold sweats, with a very low blood glucose level.

**Diagnoses:**

Considering the patient’s medical history, physical examination, and auxiliary examination, the patient was finally diagnosed with IAS.

**Interventions:**

The patient was given glucose, then the blood glucose level increased, and the symptoms were relieved. Severe hypoglycemia again occurred in the morning on the next day. Prednisone acetate tablets and glucose were given, and blood glucose monitoring was performed. The dosage of prednisone acetate was gradually adjusted in the following days.

**Outcomes:**

We combined assessments of the history of medication, identified suspicious drugs, and actively treated the patient based on this assessment. Finally, the blood glucose returned to the normal range.

**Lessons:**

This represents the first documented case of IAS triggered by the coadministration of oxaliplatin and omeprazole, addressing a critical knowledge gap regarding autoimmune hypoglycemia induced by the synergistic interaction of these 2 drug classes. Our findings offer novel insights into adverse drug reaction surveillance. For patients receiving chemotherapy in combination with proton pump inhibitors, this case highlights essential diagnostic considerations for identifying IAS as a rare complication – including the temporal relationship between hypoglycemic episodes and medication use, as well as the importance of insulin autoantibody testing. These observations may enhance clinical awareness of atypical hypoglycemia etiologies.

## 1. Introduction

Insulin autoimmune syndrome (IAS) represents a rare endocrine disorder characterized by spontaneous hypoglycemic episodes resulting from high-titer insulin autoantibodies in patients without prior exposure to exogenous insulin or insulin secretagogues.^[1]^ Currently, no established clinical guidelines exist for this condition, and its long-term prognosis remains poorly characterized. Importantly, delayed diagnosis and inadequate management may lead to refractory glycemic instability. Notably, emerging case reports highlight the underrecognized association between IAS and certain medications that are neither classic thiol drugs nor overtly contain thiol groups, posing diagnostic challenges in clinical practice.

We present a case of IAS triggered by the concomitant administration of oxaliplatin and omeprazole. Based on the pharmacological properties of these agents and their potential to generate occult thiol-containing metabolites in vivo, we conducted a thorough analysis of the possible mechanisms underlying IAS development. Additionally, we performed a comprehensive literature review to systematically compile and structurally classify all medications potentially associated with IAS pathogenesis. These findings offer novel insights into the pathophysiological mechanisms of IAS induced by atypical thiol drugs (or drugs with hidden thiol groups). This study holds significant clinical relevance for enhancing physicians’ diagnostic capabilities in identifying the causes of unexplained hypoglycemia and for optimizing pharmacovigilance systems regarding drug-induced adverse effects.

## 2. Patient information

The patient was a 70-year-old male with no significant medical history, including no family history of malignancy or relevant genetic predisposition. On November 23, 2021, he underwent laparoscopic partial rectosigmoid resection at our institution, with satisfactory postoperative recovery. Adjuvant chemotherapy was initiated on December 22, 2021, consisting of 8 cycles of mFOLFOX6 regimen combined with bevacizumab. The patient was subsequently admitted for his 9th cycle of antitumor therapy, during which no significant treatment-related adverse events were observed.

## 3. Clinical findings

The patient exhibited recurrent episodes of spontaneous hypoglycemia (venous glucose < 2.8 mmol/L) with irregular temporal patterns. Biochemical analysis during hypoglycemic events demonstrated marked elevation of the insulin release index, while counter-regulatory hormone levels (including growth hormone and adrenocortical hormones) maintained within physiological ranges. Laboratory investigations showed normal insulin-like growth factor-1 (IGF-1) concentrations but detected strongly positive insulin autoantibodies (IAA). Subsequent multimodal imaging evaluation, including PET-CT, octreotide scintigraphy, and contrast-enhanced pancreatic MRI, effectively excluded insulinoma or other insulin-secreting neoplasms, ultimately confirming the diagnosis of IAS.

## 4. Timeline

After the patient was admitted to the hospital, on June 5, 2022, he experienced morning hypoglycemia accompanied by altered mental status. Management included continuous intravenous glucose infusion supplemented with oral glucose administration to maintain euglycemia. Beginning day 6, the patient demonstrated recurrent morning hypoglycemia (blood glucose < 2.8 mmol/L), occasionally presenting with palpitations and diaphoresis, requiring symptomatic intervention. The patient received Grade I nursing care with capillary blood glucose monitoring and comprehensive diagnostic workup.

On June 8, 2022, the patient presented with persistent morning hypoglycemia, which was alleviated after treatment with a combination of oral glucose and intravenous rehydration. Laboratory findings revealed elevated C-peptide levels and significantly increased insulin autoantibody (IAA) titers, suggesting probable IAS.

On June 16, 2022, the patient had a typical episode that could reflect the clinical course, with specific details as follows:

06:00 am – blood glucose: 2.7 mmol/L (treated with 50% glucose solution x2 orally)

06:18 am – blood glucose: undetectable (It is too low to detect a specific result, And the patient developed mild chills as an accompanying symptom.)

06:18 am – treatment: additional 50% glucose x2 orally + 50% glucose x2 IV

06:40 am – blood glucose: 13.8 mmol/L

Complete daily glycemic profiles and associated symptoms are documented in Table 1, with supplementary laboratory parameters presented in Table 2.

**Table 1:** Blood glucose levels at different time points during admission.

Date	Time: recorded every day in 24 h; the symptom outcome of the patient at that time is in brackets. Blood glucose (mmol/L); the accompanying symptoms of the patient at that time are in brackets.				
June 7, 2022	3:55 am (cold sweat), 2.1 (GS)	4:25 am, 9.1	10:21 am, 2.0	4:52 pm, 2.0 (GS)	6:53 pm, 9.8
June 8, 2022	4:10 am, 2.1 (GS)	4:45 am, 9.1	11:30 am, 4.7	1:00 pm, 6.9	1:00 pm, 6.9
June 9, 2022	2:00 am, 2.5 (GS)	2:15 am, 7.8	7:00 am, 2.4 (GS)	7:15 am, 11.8	
June 10, 2022	1:55 am (cold sweat), 2.9 (GS)	2:10 am (relieved), 5.2	5:55 am (cold sweat), 1.6 (GS)	6:15 am (relieved), 7.7	
June 11, 2022	12:00 am (palpitation), 2.5 (GS)	12:15 am, 5.6	2:00 am, 10.8	6:00 am, 17.3	
June 12, 2022	6:00 am (cold sweat), 1.7 (GS)	6:15 am, 4.7			
June 13, 2022	4:50 am (palpitation), 2.2 (GS)	5:10 am, 6.0	6:00 am, 9.9		
June 14, 2022	6:44 am, 3.6 (GS)	9:10 am (palpitation), 2.8 (GS)	9:25 am (relieved), 5.0	4:00 pm, 9.8	10:00 pm, 7.8
June 15, 2022	4:00 am, 2.3 (GS)	6:00 am, 4.8	11:00 am (palpitation), 1.8 (GS)	11:40 am, 6.3	7:00 pm, 4.6
June 16, 2022	2:00 am, 7.7	6:15 am (too low) (GS)	6:20, (GS)		
June 17, 2022	5:59 am, 2.8 (GS)	6:39 am, 7.2			
June 18, 2022	6:00 am (palpitation), 3.9 (intake)	6:20 am, 5.4			
June 23, 2022	2:00 am, 3.5 (intake)	2:30 am, 6.0	6:00 am, 5.0		
June 24, 2022	6:00 am, 4.3				

GS is given oral or intravenous glucose.

GS = glucose.

**Table 2:** The laboratory results of the patient after admission.

Index	Date						
	June 6, 2022	June 7, 2022	June 8, 2022	June 9, 2022	June 10, 2022	June 16, 2022	June 24, 2022
Insulin (fasting pmol/L)	>2152↑	>2152↑	>2152↑	>2152↑	>2152↑	>2152↑	>2152↑
Insulin autoantibody (IAA RU/mL)	–	>400↑	>400↑	>400↑	>400↑	>400↑	>400↑
Islet cell antibody (IU/mL)	–	12.81	13.88	12.32	10.64	13.74	10.91
Insulin-like growth factor I (IGF-I ng/mL)	–	95	106	122	118	111	122

The content in parentheses is the unit of the index.

↑ indicates that the index exceeds the normal range.

IAAs = insulin autoantibodies, IGF-I = insulin-like growth factor-1.

## 5. Diagnostic assessment

The patient met the Whipple triad: hypoglycemia symptoms; blood glucose levels < 2.8 mmol/L; and symptoms rapidly relieved after eating or intravenous glucose injection, initially diagnosed as hypoglycemia. There are many reasons for hypoglycemia in this patient. The first consideration is drug-induced hypoglycemia: some drugs such as sulfonylureas can stimulate the secretion of human islets, causing a decrease in blood glucose, or immunosuppressive agents can also affect normal tissues (such as islet β cells producing insulin) while exerting anti-tumor responses, resulting in changes in blood glucose. We confirmed with the patient had no history of diabetes or exposure to hypoglycemic drugs or immunosuppressive drugs, which can exclude hypoglycemia caused by these factors. Pancreatic β cell in patients with insulinoma secrete a large amount of insulin into the blood, which can cause recurrent hypoglycemia.^[2]^ However, the patient ‘s octreotide scan results did not find islet cell tumors, and most of these patients exhibit negativity for insulin autoantibodies (IAAs). The patient was diagnosed with liver metastasis after sigmoid colon cancer surgery, but PET-CT showed that the liver metastases had improved, and the liver synthesis function was still acceptable, so hepatic hypoglycemia was not considered. In addition, we also try to consider tumor-induced hypoglycemia. We know that some malignant tumors secrete excessive insulin-like substances, namely, insulin-like growth factor (IGF-I and IGF-II), because IGF is structurally homologous to insulin and can interact with IGF-I and IGF-II, which are insulin receptors that exert endogenous insulin-like effects.^[3]^ However, the patient’s IGF-I results were normal, so this possibility was excluded. No insulinoma cells were detected by examination of the pancreas, and the IAA test result in the patient was positive, indicating that it was not caused by insulinoma. The patient had recurrent hypoglycemia symptoms in the morning. Considering the patient’s medical history, physical examination and auxiliary examination, the patient was finally diagnosed with IAS.

## 6. Therapeutic intervention

**Figure 1.**
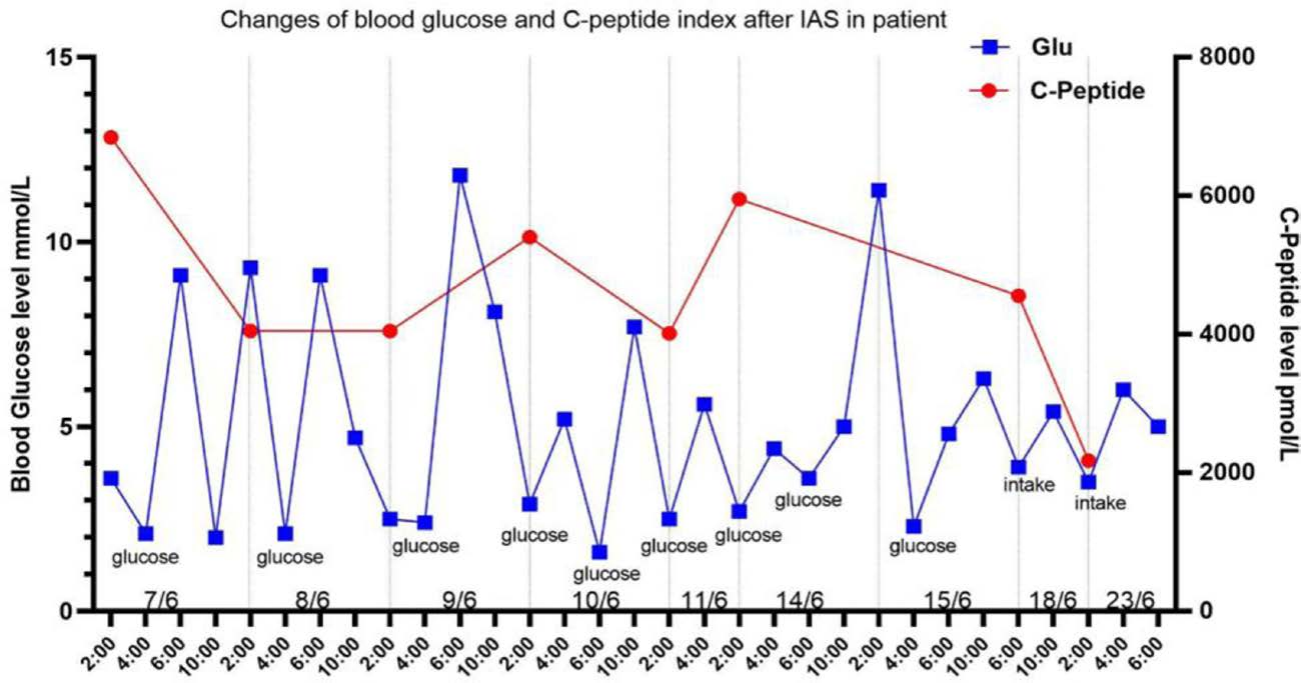
Changes of blood glucose and C-peptide results after IAS in patient. IAS = insulin autoimmune syndrome.

Beginning June 5, 2022, the patient exhibited recurrent early morning hypoglycemic episodes (blood glucose < 3.0 mmol/L), occasionally accompanied by autonomic symptoms including palpitations and diaphoresis. Therapeutic management included: continuous IV infusion of 5% or 10% glucose solutions, oral 50% glucose solution administration IV hydrocortisone sodium succinate, oral prednisone acetate tablets, oral dexamethasone acetate tablets, complete medication records are detailed in Tables 3 and 4 and Figure 1.

**Table 3:** Specific medications for patients after admission.

Date of administration	Medicine name, dosage, route of medication
June 3, 2022	Methylprednisolone (40 mg st iv); calcium folinate (700 mg st iv); fluorouracil injection (0.75 g st iv); oxaliplatin (150 mg st iv);
June 3, 2022 and June 4, 2022	Vitamin C (1 g qd iv); vitamin B6 (0.2 g qd iv); omeprazole (40 mg qd iv); granisetron (3 mg qd iv); fluorouracil injection (0.75 g st iv + 4 g st civ46h)
June 4, 2022	Bevacizumab (150 mg st iv)
June 5, 2022 to June 10, 2022	5% glucose injection (500 mL st/3 h iv)
June 5, 2022 to June 15, 2022	50% glucose injection (40 mL st po)
Multiple doses	10 % glucose injection (500 mL qd iv)
June 10, 2022 and June 11, 2022	Esomeprazole enteric capsules (20 mg qd po)

**Table 4:** Glucocorticosteroid for patients after admission.

Date	Glucocorticosteroid name, dosage, and pecific medication time
June 9, 2022	Hydrocortisone sodium succinate 100 mg
June 10, 2022 and June 13, 2022	Prednisone acetate tablets 10 mg tid (8:00 am, 12:00 pm, 5:00 pm)
June 11, 2022 and June 12, 2022	Prednisone acetate tablets 20 mg, 10 mg (6:00 am, 4:00 pm)
June 14, 2022	Prednisone acetate tablets 10 mg tid (8:00 am, 12:00 pm, 5:00 pm), 10 mg (12:00 am)
June 15, 2022	Prednisone acetate tablets 10 mg (6:00 am), 5 mg (10:00 am), 5 mg (5:00 pm), 15 mg (12:00 am)
June 16, 2022 to June 20, 2022	Prednisone acetate tablets 10 mg (6:00 am), 5 mg (10:00 am), 5 mg (4:00 pm), 15 mg + dexamethasone acetate tablets 0.75 mg (12:00 am)
June 21, 2022 to June 22, 2022	Prednisone acetate tablets 10 mg (6:00 am), 5 mg (4:00 pm), 20 mg + dexamethasone acetate tablets 0.75 mg (0:00)
June 23, 2022 to June 26, 2022	Prednisone acetate tablets 5 mg (6:00), 5 mg (4:00 pm), 25 mg + dexamethasone acetate tablets 0.75 mg (12:00 am)
June 27, 2022 to June 29, 2022	Prednisone acetate tablets 5 mg (6:00 am), 10 mg (4:00 pm), 30 mg

## 7. Follow-up and outcomes

The patient discontinued anti-tumor therapy following the hypoglycemic episodes. A 1-month postdischarge telephone follow-up confirmed no recurrence of hypoglycemia-related symptoms. The patient maintained regular blood glucose monitoring (weekly measurements), with fasting glucose levels ranging between 4 and 5 mmol/L and postprandial glucose levels between 7 and 10 mmol/L, indicating stable glycemic control.

## 8. Discussion

IAS, also known as Hirata disease, was first proposed by Japanese scholar Yukimasa Hirata and colleagues in 1970 and is an autoimmune disease characterized by hyperinsulinemia and spontaneous hypoglycemia caused by the presence of high titers of IAAs.^[4]^ IAS was originally described in Asian populations and is considered the third most common cause of hypoglycemia in Japan after insulinoma and extrapancreatic neoplasias.^[5]^ It is common in individuals older than 60 years and rare among pediatric population.^[6]^ The gold standard for the definitive diagnosis is the finding of IAA in a blood sample.^[1]^ IAS has been characterized by the combination of fasting hypoglycemia, a high concentration of total immunoreactive insulin, and the presence of antibodies to native human insulin in serum. No tissue damage has been demonstrated, which is considered a new concept for type VII hypersensitivity.^[7]^

At present, the most reported etiology of IAS involves the production of IAAs induced by external factors due to the HLA-DRB1 genotype and some autoimmune diseases (such as Graves’ disease, systemic lupus erythematosus, and systemic sclerosis).^[8]^ Viruses and drugs are the 2 most important causes. Common viruses include measles virus, mumps virus, influenza virus, Sarkozy virus and hepatitis C virus. Some scholars speculated that these viruses were used as superantigens after infection, thereby triggering the production of pathogenic IAAs.^[9]^ In addition, A 47-year-old Iranian woman with no history of diabetes, but a 10-year history of vitiligo. Considering the high level of insulin autoantibodies, the final diagnosis was IAS.^[10]^

The medications associated with IAS are mostly sulfhydryl compounds, including methimazole, lipoic acid, captopril, tiopronin, glutathione, imipenem, and acetylcysteine. The proposed mechanism involves the binding of these drugs and the cleavage of the sulfhydryl bonds between insulin chains A and B, thus leading to a conformational change in their molecular structure and promoting the increased immunogenicity of endogenous insulin. However, IAS has also been reported in patients who were taking medications that do not contain sulfhydryl bonds, such as clopidogrel, a drug with an active metabolite that contains an SH-group.^[11,12]^ Drugs without SH groups, such as albumin, loxoprofen, glucuronide, steroids and other drugs, have also been reported to cause IAS, but most of these studies are case reports, and the mechanism is not clear.

**Figure 2.**
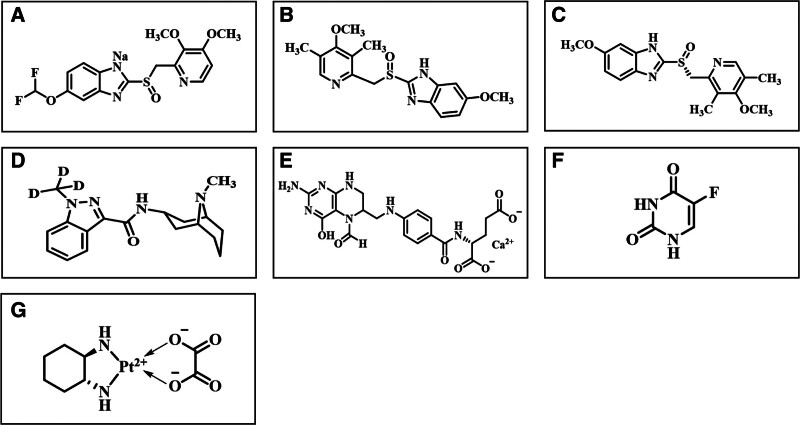
Chemical structure of major therapeutic drugs. (A) pantoprazole, (B) omeprazole, (C) esomeprazole, (D) granisetron, (E) folinic acid calcium salt hydrate, (F) 5-fluorouracil, (G) oxaliplatin.

We reviewed the structures of the drug used by the patient after admission (the chemical structure of the major therapeutic drugs is shown in Figure 2). From this perspective, omeprazole and oxaliplatin are more likely to cause IAS.

There are few cases of IAS caused by proton pump inhibitors (PPIs). Indian Gopal scholars believe pantoprazole is a precursor drug that needs to be activated in acidic environments. After being absorbed into systemic circulation, it enters gastric parietal cells and is activated to form a thiophilic sulfenamide or sulfenic acid under the catalysis of protons. This activated form reacts by covalent binding with the sulfhydryl group of cysteines and may also bind to the disulfide bond of insulin molecules, increasing immunogenicity and inducing IAS.^[13]^ The structure and functional principles of omeprazole and esomeprazole are similar to those of pantoprazole (as shown in Fig. 2). It has been reported that a 65-year-old obese Caucasian woman presented with symptomatic postprandial hypoglycemic episodes, symptom relief after carbohydrate intake and significantly elevated anti-insulin antibody levels. After the diagnosis of IAS was confirmed, her omeprazole treatment was switched to famotidine, and the IAA titer was significantly reduced.^[14]^ Another 56-year-old Chinese woman was diagnosed with IAS after taking esomeprazole for 2 weeks in 2017 with symptoms of fatigue, sweating and hunger.^[15]^ The patient had a history of chronic gastritis for 20 years and was treated with omeprazole for 1 month in 2006, during which no hypoglycemia occurred. Symptoms were relieved after discontinuation of PPIs and changes in diet. The authors speculated that the reason the patient did not exhibit IAS after previous administration of omeprazole was because esomeprazole is a levoisomer of omeprazole and that it has a lower in vivo clearance rate, higher plasma concentration and stronger acid inhibition effect than omeprazole. According to the medical history, we found that the patient had been treated with omeprazole (40 mg, qd) 8 times during chemotherapy, and IAS symptoms occurred after the ninth injection. Some scholars have speculated that drug-induced IAS does not necessarily result in hypoglycemia symptoms at the beginning of medication. Although the immune response may have occurred and IAAs were produced during the first administration of the medication, due to the low titer, it was not enough to react with a large amount of endogenous insulin. After taking the medication again, the immune response was further strengthened, and the IAA titer increased, and IAAs were able to combine with enough endogenous insulin for IAS to be detected. Under certain conditions, the dissociation of insulin and its antibody would lead to hypoglycemia.^[16]^ The reason for IAS induced by omeprazole for half a year in this patient is not clear and needs further study. For the treatment of IAS, doctors used the combination of glucose and prednisone acetate to control symptoms, and the patients gradually improved. One patient with IAS caused by methimazole was also recommended was also recommended to consume a low-carbohydrate, high-protein diet with small frequent meals to ameliorate hypoglycemic episodes.^[17,18]^

**Figure 3.**
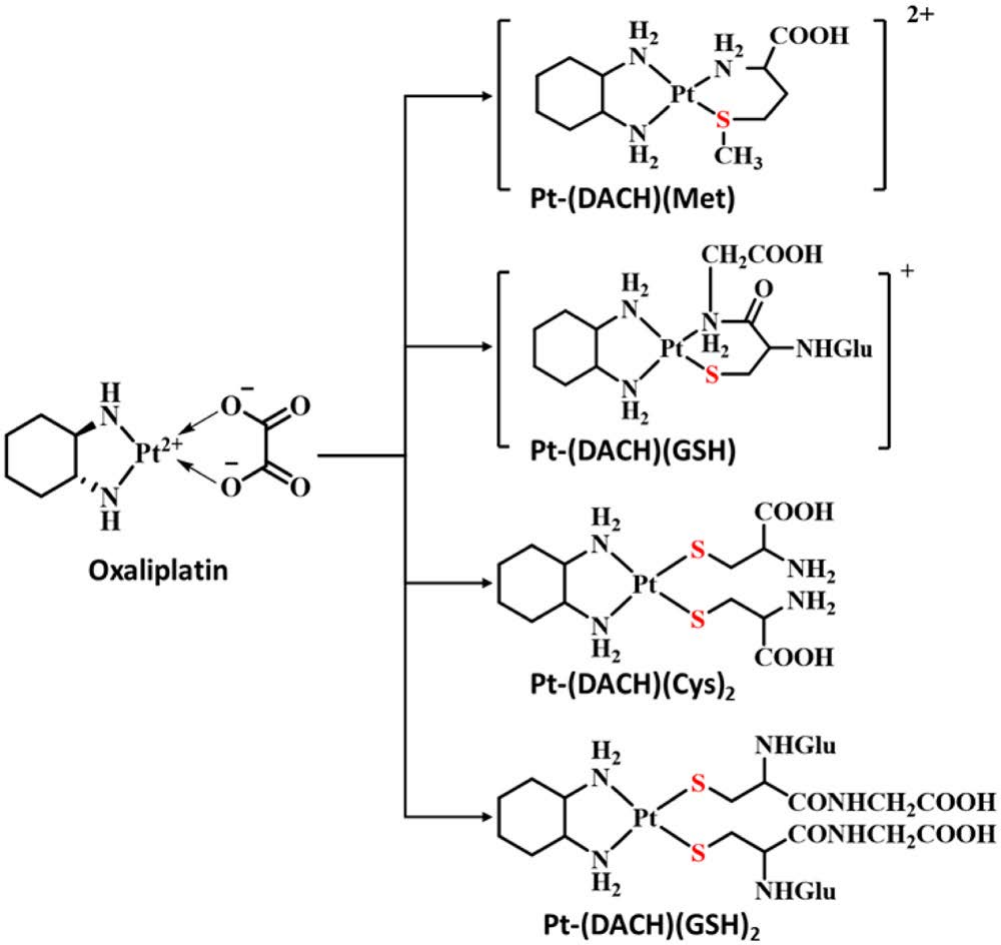
Structure of oxaliplatin and its transformation products in vivo.

Oxaliplatin is a third-generation platinum anticancer drug and is a compound of 1,2-diaminocyclohexane platinum (DACH-Pt). Its chemical structure and in vivo transformation are shown in Figure 3. Following oxaliplatin administration, platinum binding to plasma proteins and red blood cells (RBCs) is extensive and rapid (40% bound to RBCs, 33% to plasma proteins).^[19]^ A total of 27% of platinum remaining free in the plasma can be converted into platinum monochloro, dichloro or diaquo DACH. These compounds can react with cysteine (Cys), reduced glutathione (GSH) and methionine (Met), among many other substances. Thus, in vivo biotransformation products also include sulfur-containing Pt-DACH complexes, such as Pt-(DACH) (Cys)_2_, Pt-(DACH) (GSH), Pt-(DACH) (GSH)2, Pt-(DACH) (Met), and free DACH.^[20,21]^ It should be noted that the in vivo metabolic process of oxaliplatin has been confirmed by existing studies. We speculate that oxaliplatin may induce the occurrence of IAS after being converted into thiol compounds in vivo, but this hypothesis has not been confirmed by relevant literature reports. Therefore, this conclusion is only a reasonable deduction based on the existing metabolic data, which needs to be further verified by further research.

Since 1970, the number of reported cases of IAS have increased each year, and studies have shown that the incidence of drug-induced IAS may be underestimated, with the actual incidence exceeding 80%. We have summarized these structures of drugs associated with IAS in Table 5. Including chemical structures containing sulfhydryl group (tiopronin, glutathione, penicillamine and captopril), containing sulfur (imipenem, penicillin G, diltiazem, clopidogrel etc) and other classes (loxoprofen, isoniazid, tolperisone, etc).^[22–34]^

**Table 5:**
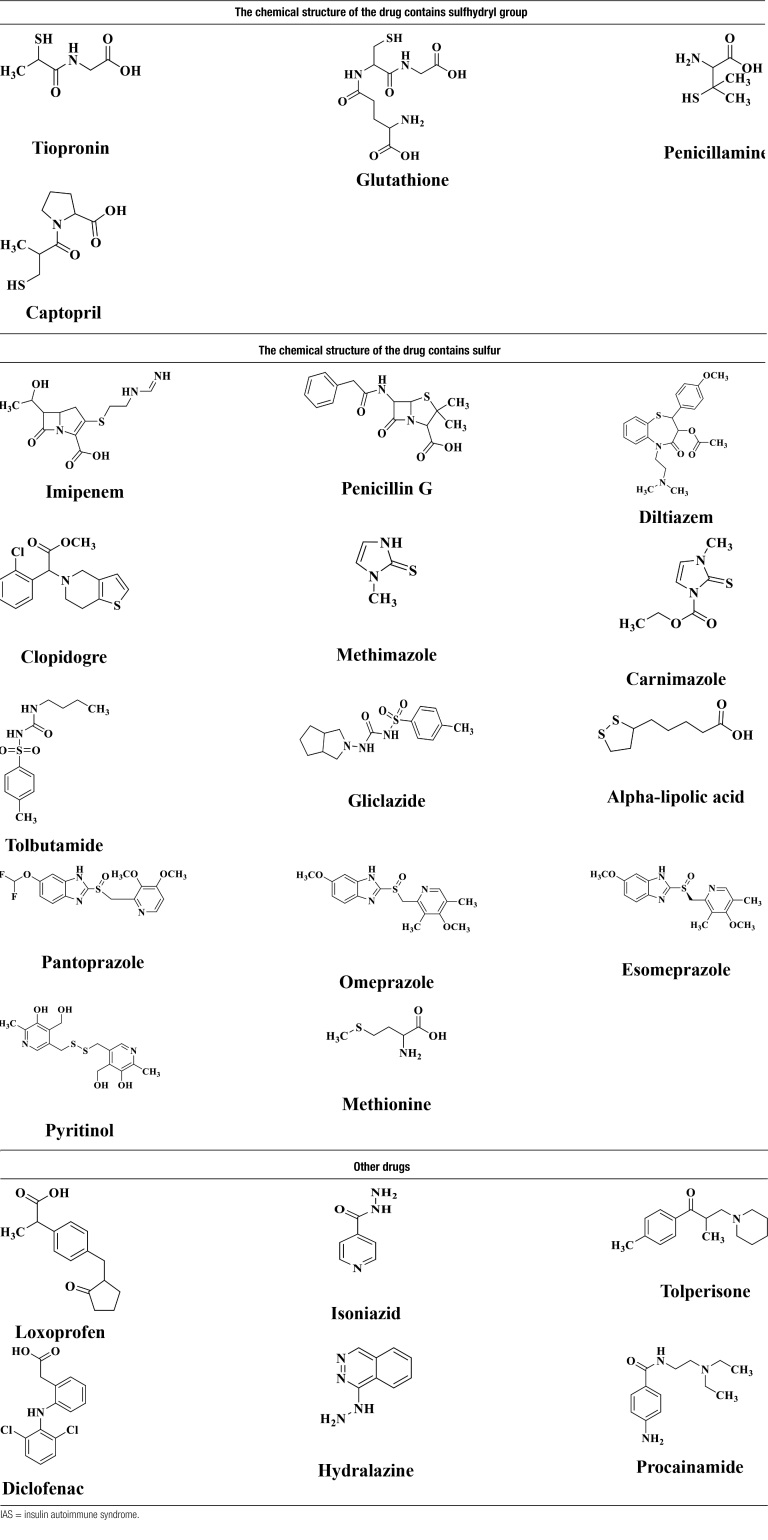
The structures of the drugs associated with IAS.

This is the first reported case of IAS induced by the combination of oxaliplatin and omeprazole, which fills the research gap in autoimmune hypoglycemia caused by the synergistic effect of these 2 types of drugs and provides a new perspective for monitoring adverse drug reactions. For the population using chemotherapy combined with PPIs, it prompts the key points for identifying IAS as a rare complication (such as the temporal correlation between hypoglycemic episodes and drug use, and the necessity of IAAs detection), which helps to improve clinicians’ vigilance against atypical causes of hypoglycemia.

Nonetheless, these results must be interpreted with caution and a number of limitations should be borne in mind. This article is only a case report, and caution should be exercised when extrapolating the conclusions. The molecular mechanism of drug-induced IAS, such as the interaction between oxaliplatin metabolites and insulin, is only inferred based on existing literature, with insufficient direct evidence. Genetic background is missing: HLA genotype detection was not performed, HLA-DRB1*0406 was originally supposed to be also associated with the alpha-lipoic acid-induced IAS in Japanese patients, but this finding was not confirmed nor in Caucasians and other Asian populations.^[35]^ It suggesting that the genetic spectrum of the syndrome may be broader, and thus indicating the need of further studies to understand more deeply the genetic background of the syndrome.^[1]^

## 9. Conclusion

In summary, we reported a case of drug-induced IAS. PPIs and oxaliplatin are widely used in clinical practice. The mechanism of the induction of IAS by these drugs is unclear. Whether they act alone or synergistically to lead to IAS remains to be further confirmed. In daily work, doctors with patients with hypoglycemia should be alert to the occurrence of IAS, and it is necessary to complete the relevant examination in time, make a clear diagnosis, and prevent the occurrence of adverse events.

## Author contributions

**Data curation:** Shuowen Wang.

**Investigation:** Guorong Fan.

**Resources:** Guorong Fan.

**Validation:** Leilei Bao.

**Writing – original draft:** Xianglei Wu.
